# Evidence-Based Treatment of *Pseudomonas aeruginosa* Infections: A Critical Reappraisal

**DOI:** 10.3390/antibiotics12020399

**Published:** 2023-02-16

**Authors:** Arta Karruli, Christian Catalini, Chiara D’Amore, Francesco Foglia, Fabio Mari, Arjan Harxhi, Massimiliano Galdiero, Emanuele Durante-Mangoni

**Affiliations:** 1 Department of Precision Medicine, University of Campania ‘Luigi Vanvitelli’, 80138 Naples, Italy; 2 Department of Infectious Diseases, University Hospital “Mother Teresa”, 10001 Tirana, Albania; 3 Department of Advanced Medical and Surgical Sciences, University of Campania ‘Luigi Vanvitelli’, 80138 Naples, Italy; 4 Infectious Diseases Unit, San Giovanni di Dio e Ruggi D’Aragona Hospital, 84131 Salerno, Italy; 5 Unit of Microbiology and Virology, Department of Experimental Medicine, University of Campania ‘Luigi Vanvitelli’, 80138 Naples, Italy; 6 Department of Emergency Medicine, University “Federico II”, 80138 Naples, Italy; 7 Unit of Infectious and Transplant Medicine, AORN Ospedali dei Colli-Monaldi Hospital, 80131 Naples, Italy

**Keywords:** *Pseudomonas aeruginosa*, resistance, treatment, multidrug-resistant bacteria, extensively drug resistant

## Abstract

Multidrug-resistant (MDR)/extensively drug-resistant (XDR) *Pseudomonas aeruginosa* is emerging as a major threat related to adverse patient outcomes. The goal of this review is to describe evidence-based empiric and targeted treatment regimens that can be exploited when dealing with suspected or confirmed infections due to MDR/XDR *P. aeruginosa*. *P. aeruginosa* has inherent resistance to many drug classes, the capacity to form biofilms, and most importantly, the ability to quickly acquire resistance to ongoing treatments. Based on the presence of risk factors for MDR/XDR infections and local epidemiology, where large proportions of strains are resistant to classic beta-lactams, the recommended empirical treatment for suspected *P. aeruginosa* infections is based on ceftolozane-tazobactam or ceftazidime-avibactam. Where local epidemiology indicates low rates of MDR/XDR and there are no risk factors, a third or fourth generation cephalosporin can be used in the context of a “carbapenem-sparing” strategy. Whenever feasible, antibiotic de-escalation is recommended after antimicrobial susceptibility tests suggest that it is appropriate, and de-escalation is based on different resistance mechanisms. Cefiderocol and imipenem-cilastatin-relebactam withstand most resistance mechanisms and may remain active in cases with resistance to other new antibiotics. Confronting the growing threat of MDR/XDR *P. aeruginosa*, treatment choices should be wise, sparing newer antibiotics when dealing with a suspected/confirmed susceptible *P. aeruginosa* strain and choosing the right option for MDR/XDR *P. aeruginosa* based on specific types and resistance mechanisms.

## 1. Introduction

*Pseudomonas aeruginosa* is a Gram-negative rod commonly associated with nosocomial infections. In the 2017 global priority list of pathogens, the World Health Organization (WHO) ranked *Pseudomonas aeruginosa* in the category of highest priority [1]. It is also part of the ESKAPE pathogens, a set of six microorganisms (*Enterococcus faecium*, *Staphylococcus aureus*, *Klebsiella pneumoniae*, *Acinetobacter baumannii*, *Pseudomonas aeruginosa*, and *Enterobacter* spp.) with peculiar features in terms of increasing resistance patterns; indeed, ESKAPE are not only growing in terms of the quantity of resistance, i.e., increasing incidence, but also due to quality because of the development of new resistance mechanisms [2]. *Pseudomonas aeruginosa* was the fifth most common cause of hospital-acquired infections (HAI) in a point prevalence study from 28 European countries in 2016–2017, with a prevalence of 7.1%. In this study, HAI had an overall prevalence of 6.8%, of which 33.1% were attributed to multidrug-resistant (MDR)/extensively drug-resistant (XDR) pathogens [3]. The prevalence *of P. aeruginosa* may be up to 23% in patients with intensive care unit (ICU)-acquired infections [4] and the prevalence of resistant *P. aeruginosa* may reach 48.7% in the ICU [5]. As reported by the U.S. Centers for Disease Control and Prevention (CDC), 32,600 hospitalized patients were infected by MDR *P. aeruginosa* in 2017 [6].

MDR/XDR *P. aeruginosa* has become a major threat related to healthcare with negative consequences in terms of increased mortality, morbidity, and healthcare costs [7,8,9]. The treatment of these infections remains challenging and requires top skills in the context of antimicrobial stewardship programs.

Accordingly, the goal of this review is to critically reappraise the most recent available evidence in order to describe potential empiric and targeted treatment regimens that can be used for *P. aeruginosa* infections that are suspected or confirmed to be MDR/XDR. We also summarize de-escalation strategies based on antimicrobial susceptibility results and the mechanisms of resistance.

## 2. Search Strategy and Design of the Review

The authors conducted an extensive literature review by utilizing the MEDLINE/Pubmed and Cochrane library databases and searching for articles regarding *P. aeruginosa* epidemiology, infection syndromes, resistance mechanism, diagnosis, treatment, both empirical and targeted (definitive) regimes, and outcomes. In order to better put into context the data on treatment choices, we also briefly recap the most important microbiological features of *Pseudomonas aeruginosa*. The search terms included a combination of the word *P. aeruginosa*/MDR *P. aeruginosa* in addition to one of the following: “treatment”, “risk factors”, “biofilm”, “new beta lactams”, ” antimicrobial susceptibility testing”, and “resistance mechanisms”. While we searched for studies regardless of their language, only studies reported in English were included.

## 3. *Pseudomonas aeruginosa* Information Path: Walking in the Right Direction

### 3.1. Resistance Patterns and Infection Syndromes

As per Magiorakos’s definition, MDR *Pseudomonas* is defined as being not susceptible to at least one antibiotic in at least three antibiotic classes to which it is usually susceptible, while XDR *Pseudomonas* is defined when there is non-susceptibility to at least one antimicrobial agent in all but two or fewer antimicrobial classes [10]. In 2018, a new concept of “difficult-to-treat resistance” (DTR) was introduced [11]. DTR is defined as *P. aeruginosa* exhibiting non-susceptibility to all of the following: ceftazidime, cefepime, piperacillin-tazobactam, imipenem-cilastatin, meropenem, ciprofloxacin, levofloxacin, and aztreonam. According to data published by the European Centre for Disease Prevention and Control (ECDC) in 2020, 30.1% were resistant to at least one antibiotic among carbapenems, fluoroquinolones, ceftazidime, piperacillin-tazobactam, and aminoglycosides, whereas 17.3% were resistant to two or more antibiotics [12].

**Figure 1:**
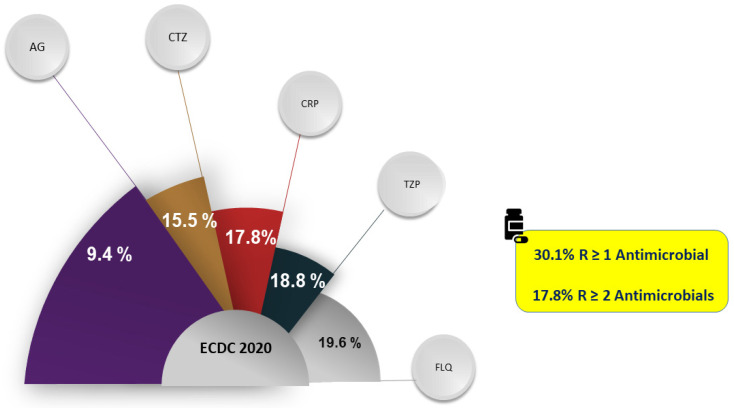
Prevalence of *Pseudomonas aeruginosa* resistance to common antimicrobials/classes in Europe. Abbreviations: AG, aminoglycosides; CTZ, ceftazidime; CRP, carbapenems; FLQ, fluoroquinolones; TZP, piperacillin-tazobactam; R, resistant, ECDC, European Centre for Disease Prevention and Control; R, resistant [12].

As observed, 9.4% of the isolates from 29 European countries were resistant to aminoglycosides, 15.5% were resistant to ceftazidime, 17.8% were resistant to carbapenems, 18.8% were resistant to piperacillin-tazobactam, and 19.6% were resistant to fluoroquinolones [12] (Figure 1). Countries with a higher prevalence of *P. aeruginosa* were also those with the highest prevalence of Gram-negative resistance, probably due to shared risk factors.

**Figure 2:**
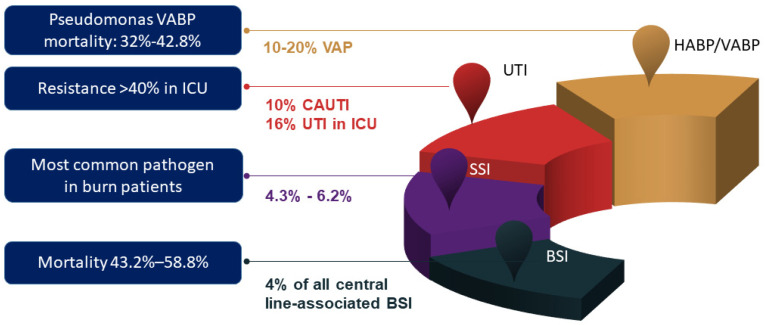
Most common healthcare–associated infections caused by *P. aeruginosa* [13]. Abbreviation: BSI, bloodstream infection; CAUTI, catheter-associated urinary tract infection; UTI, urinary tract infection; ICU, intensive care unit; HAP, hospital-acquired pneumonia; SSI, surgical site infection, VAP, ventilator-associated pneumonia.

Usually, the most common infections due to PA are respiratory tract infections, including hospital-acquired pneumonia (HAP)/ventilator-associated pneumonia (VAP), urinary tract infections (UTI), bloodstream (BSI), and skin and soft tissue infections. The most common types of *P. aeruginosa* infection are lower respiratory tract infections; it has a prevalence of 10–20% in VAP, which is the second most common pathogen after *S. aureus*. Mortality in VAP and bloodstream infections due to *P. aeruginosa* may be as high as 40% [13] (Figure 2). *P. aeruginosa* is the most common cause of otitis externa and keratitis and is also a common pathogen in diabetic foot infections and endocarditis [14,15,16].

### 3.2. P. aeruginosa Diagnosis

#### 3.2.1. Planktonic form: Bacterial Identification and Antibiotic Susceptibility Testing

Samples presumptively positive for *P. aeruginosa* are grown and developed on a routine basis from a MacConkey medium; if they exhibit lactose non-fermenting, pale colonies and are oxidase-positive, they can be considered suspects for *P. aeruginosa*. Suspected colonies can be rapidly identified using matrix-assisted laser desorption/ionization time of flight (MALDI-TOF). Antimicrobial susceptibility tests (ASTs) can be performed using disk diffusion (Kirby–Bauer) and broth microdilution (BMD) according to current guidelines of the European Committee on Antimicrobial Susceptibility Testing (EUCAST) or Clinical and Laboratory Standards Institute (CLSI) [17,18]. Moreover, AST can be performed using automated systems, such as Vitek 2, or gradient tests, including E-tests. Typical antibiotics tested are grouped into different categories: β-lactams (ceftazidime, cefepime, piperacillin-tazobactam, and aztreonam); fluoroquinolones (levofloxacin and ciprofloxacin); aminoglycosides (amikacin, gentamicin, and tobramycin); carbapenems (imipenem-cilastatin, meropenem, and doripenem); and colistin [19]. Resistance should be defined as when an isolate is resistant to an antibiotic to which it was previously susceptible. The strains with intermediate susceptibility to antimicrobial agents are now considered susceptible if an elevated exposure to the antibiotic can be attained. Based on the AST results, *P. aeruginosa* should be classified into carbapenem-resistant, fluoroquinolone-resistant, aminoglycoside-resistant, cephalosporin-resistant, and piperacillin/tazobactam-resistant, using the most current standard definitions, or *phenotypes* (Table 1) [20].

Regarding new antibiotics, a study on 200 *P. aeruginosa* isolates assessed the efficacy of the Vitek-2 automatic system, disk diffusion, and gradient tests in detecting antibiotic resistance in comparison to the gold standard BMD, and the study showed that ceftazidime/avibactam disk diffusion and gradient tests had a good performance, whereas ceftolozane-tazobactam gradient tests performed better compared to disk diffusion and Vitek-2 [21]. For meropenem-vaborbactam, both Etest and Vitek-2 proved to be accurate [22,23]. BMD remains the gold standard for Cefiderocol as gradient strips are discouraged and many cefiderocol-resistant isolates may fail the diagnosis with disc diffusion [24]. Moreover, AST for colistin, a classic antibiotic used to treat *Pseudomonas* spp. infections, is usually carried out with BMD due to the low accuracy of automated systems and poor diffusion in disk and gradient tests, which underestimate the MIC of colistin, thereby overestimating colistin activity [25,26].

In 2019, a new concept of “intermediate susceptibility” was introduced as being susceptible with increased drug exposure. This means that with the optimization of PK/PD parameters, increased doses, or the optimized mode of antibiotic administration, a microorganism could be considered susceptible to that antibiotic despite the MIC ranging between S and R breakpoints according to EUCAST criteria [27].

Recently, EUCAST has defined new breakpoints for susceptibility according to MIC for *P. aeruginosa.* Most antibiotic classes have an arbitrary breakpoint <= 0.001 mg/L (which is generally lower than the actual MIC of most agents) classifying all these antibiotics as susceptible with increased exposure. In Table 2, we list antipseudomonal antibiotics and current MIC breakpoints according to EUCAST 2023 compared to 2019 EUCAST criteria when the new definition was introduced [28]. The antibiotic classes affected by this change are mostly beta lactams, including ceftazidime, cefepime, piperacillin-tazobactam, aztreonam, imipenem-cilastatin, and fluoroquinolones such as ciprofloxacin and levofloxacin. They are now mostly “susceptible with increased exposure” since their actual MIC in clinical practice is always higher than the susceptibility breakpoint.

Further tests such as molecular and PCR-based methods can be applied in diagnoses to obtain early results, including the identification of the implied pathogens, or to investigate the resistance profiles of specific isolates; this method can be used to detect the presence of specific resistance enzymes such as Ambler class C beta-lactamases (AmpC), *Klebsiella pneumoniae* carbapenemase (KPC), New Delhi Metallo-ß-lactamase (NDM), Verona Integron-encoded Metallo-β-lactamase (VIM), imipenemases (IMP), Guiana extended-spectrum β-lactamase (GES), or oxacillinases (OXAs). 

#### 3.2.2. Sessile form: Biofilm Detection

All mentioned methods use the planktonic (free-living) form of *P. aeruginosa* bacteria. Biofilm-forming bacteria have in contrast different resistance characteristics compared to their planktonic counterpart in terms of different architectures of bacterial cooperation, gene expression, and biochemical activity [29]. Biofilm is a never-ending cycle composed of more than one type of bacteria/microbe organized in sessile forms covered by a matrix of extracellular polymeric substances (EPS) [30]. As observed, 90% of the *P. aeruginosa* biofilm is composed of a matrix of polysaccharides, extracellular DNA (eDNA), proteins, and lipids, making an efficient barrier to antibiotic entry [31,32,33]. Biofilm is known as “the city of microbes” [34], with the matrix being the “house of the biofilm cells” [35]. When bacteria switch to biofilm growth types, sessile forms may undergo major phenotypic changes [36,37,38], with an increase in the gene expression of efflux pumps, cell wall components, and peptidoglycan synthesis [39,40]. In fact, it was shown that the MIC of different antibiotics within biofilms may increase by 10–1000-fold [41].

Biofilm production is also implicated in multidrug-resistant *P. aeruginosa* (MDR-PA). A study showed that cephalosporins and carbapenems and biofilm-producing *P. aeruginosa* exhibited a 50% susceptibility rate compared to the non-biofilm-producing strains showing a 100% and 81.8% sensitivity, respectively [42]. Biofilm was shown to affect antimicrobial resistance also in other types of bacteria [43,44]. Various methods can be used to detect biofilm formation in *P. aeruginosa*, with some having a better performance than others [45,46]. Some tests are time-consuming, and omic tools may require qualified technical expertise [47]. However, from tube adherent to microtiter assays, MALDI-TOF and omic analyses are not part of standardized protocols [47,48]. Moreover, currently, the implementation of standardized breakpoint values to assess the MIC of different antibiotics within biofilms remains an unmet need [47].

### 3.3. Risk Factors for P. aeruginosa/Resistant P. aeruginosa Infection

Risk factors for *P. aeruginosa* infections are burns, open wounds, and post-surgery status for soft tissue infections; urinary catheter for urinary tract infections; immune compromise for bloodstream infections; and old age, chronic obstructive pulmonary disease (COPD), cystic fibrosis, and mechanical ventilation for respiratory infections [49]. 

For respiratory infections, factors associated with *P. aeruginosa* community-acquired pneumonia (PA-CAP) were prior PA colonization/infection, prior tracheostomy, bronchiectasis, severe COPD, and prior invasive respiratory or vasopressor support (IRVS), while factors associated with MDR-PA CAP were tracheostomy, previous colonization/infection, and IRVS [50]. The longer duration of hospitalization/ICU stay was associated with VAP due to *P. aeruginosa* [51,52].

Regarding resistant isolates, various studies have tried to identify risk factors for MDR *P. aeruginosa* acquisition. A systematic review of 28 articles evidenced that the development of MDR isolates was associated with prior antibiotic use and prior hospitalization or ICU stay [53]. Another systematic review of 22 studies in Asia-Pacific countries evidenced previous exposure to antimicrobials, mechanical ventilation, and previous hospitalization as risk factors for *P. aeruginosa* infections. Risk factors for MDR isolates included mechanical ventilation, previous hospitalization, diabetes mellitus, surgery, prolonged hospital stay, and higher Acute Physiology and Chronic Health Evaluation (APACHE) II score [54].

Other studies identified ICU stay, bedridden state, having high invasive devices scores, being treated with broad-spectrum cephalosporins and with aminoglycosides, mechanical ventilation, higher severity index score, previous hospitalizations, and co-morbidities (diabetes mellitus, renal failure, COPD, and cystic fibrosis) as significant risk factors for MDR *P. aeruginosa* carriage [55,56,57,58,59]. Similar factors were seen also in other resistant Gram-negative infections [60,61,62]. Moreover, the factors associated with the presence of *P. aeruginosa* infections are broadly similar to those associated with MDR-PA [49,50,63,64] probably due to the nosocomial nature of most PA infection acquisitions [65].

### 3.4. Major Resistance Mechanisms

*P. aeruginosa* exerts its resistance by possessing inherent one-to-many drug classes [66,67], the ability to quickly acquire resistance to ongoing treatments, and the capacity to form biofilm [68]. The known mechanisms of PA resistance include intrinsic resistance: outer membrane permeability, overexpression of efflux systems, and antibiotic-inactivating enzymes; acquired resistance: horizontal gene transfer and mutations to genes encoding for efflux pumps, porins, penicillin-binding proteins, and enzymes; and adaptive resistance: continuous antibiotic exposure and overexposure to environmental stress [69]. 

The mechanisms of action of classic antipseudomonal antibiotics are numerous: bacterial cell wall inhibition for beta-lactam agents such as ceftazidime/cefepime, piperacillin-tazobactam, imipenem-cilastatin, meropenem, doripenem, and aztreonam; blockage of DNA synthesis for fluoroquinolones; and protein synthesis inhibition for aminoglycosides [69]. The major mechanisms of resistance of *P. aeruginosa* are described in Table 3 [66,68,69,70,71,72,73].

Multiple mechanisms of resistance often coexist and cooperate to confer *P. aeruginosa* resistance to multiple antimicrobials, thus contributing to challenging treatment efforts [74].

### 3.5. Treatment

#### 3.5.1. Empirical Treatment

Empirical treatment should be initiated as soon as cultures are collected, as early (and appropriate) therapy is associated with a better prognosis [75] and mostly so in patients with sepsis or septic shock. 

Empirical treatment is usually based on the presence of risk factors and local epidemiology for MDR-PA [76,77]. In patients hospitalized in settings where the local epidemiology suggests an MDR-PA rate lower than 25% and without risk factors for MDR-PA, treatment includes one antipseudomonal antibiotic, such as, in decreasing order of priority, carbapenem; piperacillin-tazobactam; cefepime; ceftazidime in cases of BSI, VAP, and SSTI; and all of the above as well as aminoglycosides or colistin in cases of complicated UTI [77]. In patients hospitalized in settings where the local epidemiology suggests an MDR-PA rate higher than 25% and/or the presence of risk factors for MDR-PA/Gram-negative pathogens or in critically ill patients, empirical treatment should include newer beta-lactams ceftolozane-tazobactam, ceftazidime-avibactam, imipenem-cilastatin-relebactam, or a combination of classic antipseudomonal agents plus an aminoglycoside, colistin, or fosfomycin [77] where new antibiotics are not available. 

#### 3.5.2. Combination Therapy or Monotherapy

Regarding combination therapy versus monotherapy, various studies did not demonstrate the superiority of combination therapy compared to monotherapy as a definitive treatment in terms of mortality, microbiological eradication, or resistance development [78,79,80]. In a study of 1119 patients with bacteremia due to PA, combination treatments did not show lower mortality compared with monotherapy [80]. A systematic review of 69 studies comparing a combination of beta-lactams with aminoglycosides to monotherapy did not show lower rates of resistance development with combination treatment, and in addition, adverse events such as nephrotoxicity were more common in the combination group [78]. However, in patients with COPD exacerbation, combination therapies with fluoroquinolones proved to be superior in terms of microbiological eradication and mortality [79]. Moreover, when studied in the context of initial empirical treatments, inadequate empirical antibiotic therapy was shown to be associated with increased mortality, whilst adequate combination therapies translated into decreased mortality [81]. Furthermore, one study evidenced that targeted combination treatments with ciprofloxacin correlated with lower rates of mortality compared to monotherapy [82]. The reason for choosing a combination therapy lies in the increased chances that the isolate may be susceptible to at least one of the chosen antibiotics. This applies mostly to classic beta-lactam agents. Regarding new antibiotics, ceftolozane-tazobactam, ceftazidime-avibactam, or imipenem-cilastatin-relebactam monotherapy is preferred over combination therapy [83]. One option to exploit possible synergistic effects without adding excessive toxicity would be to limit combination therapy, e.g., with an active aminoglycoside, to a short course (maximum 2 to 5 days), with early de-escalation applied to an active monotherapy. Indeed, it was shown that the short course combination of aminoglycosides with beta lactams contributed to synergistic bacterial killing phenomenon at 24 h and lower rates of resistance development probably due to the different mechanisms of action. These antibiotics have no common efflux pump resistance and the cell wall disruption caused by beta lactams may increase the target concentration/penetration of aminoglycosides [84,85] However, this short course combination did not show a reduction in mortality in BSI due to Gram-negative bacteria, including *P. aeruginosa* [86].

In any case, high doses of drugs should always be used, particularly at the outset, and schedules should be adapted to the molecule’s pharmacokinetic properties.

#### 3.5.3. Definitive Treatment after AST Results

Once AST results are available, treatment should be individualized/simplified by choosing the most effective antibiotic with the narrowest spectrum of activity. For MDR-PA, a carbapenem or a new beta-lactam agent, where possible with a carbapenem-sparing strategy, should be administered. 

Here, we describe the possible scenarios of PA treatments starting from the wild-type PA, which is susceptible to all antipseudomonal antibiotics, to DTR-Pseudomonas, which is resistant to all classical antipseudomonal agents [87,88].

##### Wild-Type *Pseudomonas aeruginosa*

Despite being the most susceptible form of this microorganism, wild-type *Pseudomonas* has intrinsic resistance to various antibiotics. Its common expression of an inducible AmpC cephalosporinase, usually at low levels, plus efflux systems and low membrane permeability confer the intrinsic resistance of PA to first and second generation cephalosporins, some of the third generation cephalosporins (such as cefotaxime and ceftriaxone), and ertapenem. Interestingly, rather than showing intrinsic resistance to an entire class of antibiotics, PA often shows resistance to individual antibiotics within a given class. As such, the preferable antibiotics in descending order of importance are ceftazidime as first choice (showing the narrowest spectrum of activity compared to cefepime and piperacillin-tazobactam) followed by cefepime and piperacillin-tazobactam. Fluoroquinolones are a valid option in cases when oral treatment can be initiated in an outpatient setting, e.g., in skin and soft tissue infections or in cases of patients that are discharged and need continuing treatment at home after discharge. Carbapenems are the last option used in order to preserve them from more difficult infections. [89,90]. Regarding fluoroquinolones, even though they are the only anti-pseudomonal agents with an oral formulation, they also have important adverse effects in inducing resistance, particularly efflux pump overexpression. Indeed, this mechanism also confers resistance to other antibiotics that may have not been used by that particular patient [91]. Specifically, efflux-system-based resistance is one of the most important resistance mechanisms of PA, involving a variety of antibiotics including quinolones, aminoglycosides, and beta-lactams such that the use of a single agent may trigger cross-resistance to other agents that are susceptible to that resistance mechanism [77]. As mentioned above, most published studies did not show a superior combination treatment in cases of definitive treatments. However, a question mark remains regarding empirical treatment, where combination treatment increases the chance of using at least one active (and possibly effective) antibiotic. Therefore, in cases of definitive treatments, other antibiotics such as aminoglycosides, colistin, or fosfomycin remain as alternatives to backbone agents (i.e., for cases experiencing side effects of classic antipseudomonal agents or with strains resistant to the latter). A more comprehensive discussion of this point follows.

##### Specific Antibiotic Class, Possible Applications, and Resistance Scenarios

Aminoglycosides

Amikacin, gentamicin, and tobramycin are the aminoglycosides used for *P. aeruginosa* infections. Regarding resistance, the most common mechanism is via antibiotic inactivation by aminoglycoside-modifying enzymes, resulting in a reduction in the affinity of aminoglycosides for ribosome subunit target 30S, thus blocking their activity [77]. Amikacin is the aminoglycoside that is less susceptible to this mechanism [92]. However, these antibiotics should not be used as a monotherapy in infections outside the urinary tract [82,92]. Apart from i.v formulation, other forms have been shown to be effective, such as inhaled tobramycin, which was shown to be effective in the acute exacerbation of cystic fibrosis [93]. Plazomicin, a novel aminoglycoside agent, does not overcome resistance mechanisms such as altered membrane permeability and other aminoglycoside resistance mechanisms, and its use is limited to *P. aeruginosa* urinary infections [94].

Polymixins

Colistin is a drug mostly used in MDR pathogen infections with activity also against *P. aeruginosa*. It is not a novel antibiotic, however, and its use is problematic due to its side effects, such as the increased risk of nephrotoxicity [95]. The combination of intravenous and nebulized formulations was effective and safe in treating VAP due to Gram-negative pathogens, including *P. aeruginosa* [96]. The additive effects of aerosol administration may be due to its concentrations in the epithelial lining fluid (ELF), since it was shown that the i.v. formulation does not reach an adequate concentration in ELF, whereas the aerosol compound does [97]. The empirical use of colistin in Gram-negative pathogens was not associated with higher chances of survival [98]; moreover, combination treatment with meropenem for carbapenem-resistant Gram negatives was not superior to monotherapy [99,100,101] or compared to synergizing with rifampicin [102].

Fosfomycin Disodium

Fosfomycin disodium is another old antibiotic with a unique mechanism of bactericidal activity, exerted by the inactivation of enzymes important in bacterial cell wall synthesis. Another unique characteristic of this antibiotic, compared to other anti-pseudomonal agents, is the lack of cross-resistance between fosfomycin and other antibiotics, such as beta lactams and aminoglycosides [103]. It is active against resistant isolates of *P. aeruginosa* due to the low rates of use and non-shared resistance mechanism with other antibiotics [104]. However, caution should be exercised in treating with this drug due to the possibility of the rapid development of antibiotic resistance during treatment after exposure. Fosfomycin was shown to be effective in treating PA as a combination therapy with other antipseudomonal agents in cystic fibrosis patients [105] and also as a combination treatment with carbapenems, exhibiting a synergistic effect with decreasing carbapenem MICs [106]. It was shown to be effective in monotherapy for complicated urinary tract infections compared to piperacillin-tazobactam where all patients with *P. aeruginosa* infection achieved clinical cure [107].

##### High MIC of Conventional Antipseudomonal Beta Lactams

Some PA isolates may be in vitro susceptible to beta-lactam agents but with an MIC near the breakpoint for conventional beta-lactams. In this case, a treatment solution could be the administration of a high-dose, extended infusion of classic beta lactams in order to reach the exposure needed for the right pharmacokinetic and pharmacodynamic target attainment. Such an effect can be achieved by the prolonged exposure of bacteria to a concentration of antibiotics above the MIC, exploiting in this manner the time-dependent effect of beta-lactam agents [108]. Bauer et al. evaluated 87 respiratory/bloodstream infections treated with either the intermittent or extended infusion of cefepime and found that the group given extended infusions had a lower mortality rate [109]. Moreover, piperacillin-tazobactam-extended infusion exhibited lower mortality rates and a shorter hospitalization length, translating into lower healthcare costs as well [110]. However, this treatment strategy needs to be wisely chosen due to difficulties in implementation, such as limiting patient mobility due to prolonged i.v. line infusion and protracted indwelling access of intravenous lines leading to possible infective or thrombotic complications. 

##### Resistance to Carbapenems with Maintained Susceptibility to Cephalosporins

This is a scenario mostly observed in cases with a low expression/limited production of porins (especially OprD). In these cases, isolates are resistant to carbapenems (meropenem and imipenem-cilastatin) due to porin channels being an important mechanism in the bacterial entry for carbapenems. However, the isolate may maintain susceptibility to cephalosporins. Therefore, two treatment options may be applicable: i) new beta lactams (ceftolozane-tazobactam and ceftazidime-avibactam) and (ii) the high-dose extended-infusion of classic “unprotected” cephalosporins. The decision is up to the treating clinician; however, IDSA recommends—after AST repetition and confirmation of the results—starting treatments with conventional cephalosporins in order to preserve newer antibiotics for future infections. In cases with a severe infection and in critically ill patients, the use of new beta lactams could be a more reasonable option [89].

Beta Lactam Resistance

Most common resistance mechanisms found in *P. aeruginosa* include the production of beta-lactamases, such as some ESBL but mostly the hyper-expression of AmpC, which confers resistance to ceftazidime/cefepime, piperacillin-tazobactam, and aztreonam. In this case, carbapenems remain active; thus, the treatment choice is between carbapenems and newer beta lactams. 

Carbapenem Resistance

The most common mechanism is the production of carbapenemases, particularly class A KPC or GES and metallo-beta-lactamases such as VIM, IMP, SBL, and other less common ones such as GIM, NDM, and FIM. VIM and IMP have a few variants implicated in *P. aeruginosa* resistance, whereas only one type is identified for others [111]. Metallo-beta-lactamases confer resistance to all antibiotics (including ceftolozane-tazobactam and ceftazidime-avibactam) except aztreonam. In contrast, cefiderocol is not affected [112,113]. Usually, isolates with metallo-beta-lactamase production also express AmpC. The combination of aztreonam/avibactam is therefore a promising option in such resistance settings, as aztreonam is not affected by the hydrolysis of metallo-beta-lactamases, while avibactam inhibits most other co-expressed beta-lactamases, including AmpC [114].

Isolates producing KPC (not a very common resistance mechanism in *P. aeruginosa*) are susceptible to cefiderocol and imipenem-cilastatin-relebactam. Another treatment option for carbapenemase-producing strains could be the combination of aminoglycosides, colistin, or fosfomycin with another antibiotic that is found active in vitro.

DTR *Pseudomonas aeruginosa*

Efflux systems and decreased membrane permeability are the mechanisms of resistance that confer reduced susceptibility to a wide range of anti-pseudomonal antibiotics. Indeed, efflux systems affect beta-lactams, carbapenems, fluoroquinolones, and aminoglycosides, and decreased membrane permeability mostly affects beta-lactams, fluoroquinolones, and aminoglycosides. Sometimes, different mechanisms cooperate in the same isolate—for example, the modification of OprD mostly affects imipenem-cilastatin (and to a lesser extent meropenem) but does not affect other beta-lactams. However, this mechanism is often associated with other resistance mechanisms such as efflux systems, the hyperproduction of AmpC enzymes, and the mutation of penicillin-binding proteins, making the isolate resistant to most/all conventional anti-pseudomonal antibiotics [76]. In this case, the needed therapeutic approach might be that of a DTR *P. aeruginosa.*

The concept of “difficult-to-treat resistant” (DTR) *Pseudomonas aeruginosa*, proposed in 2018, is based on the not so rare instance of a strain resistant to all of the following antibiotics: ceftazidime, cefepime, piperacillin-tazobactam, aztreonam, imipenem-cilastatin, meropenem, ciprofloxacin, and levofloxacin. IDSA divides the therapeutic approach for DTR-PA into two distinct scenarios: urinary tract infections and non-urinary tract infections. In urinary tract infections, recommended treatment choices are newer beta lactams (ceftazidime-avibactam, ceftolozane-tazobactam, imipenem-cilastatin-relebactam, and cefiderocol) as the first choice, followed by a single dose of aminoglycosides as a second choice; in complicated and non-complicated infections, the first and second choices are the same, with complicated infections requiring the addition of colistin as an alternative therapy. In infections outside of the urinary tract, the first choices are ceftazidime-avibactam, ceftolozane-tazobactam, imipenem-cilastatin-relebactam, and as alternative therapy, cefiderocol [89]. Regarding newer beta lactams, there are no available clinical trial data comparing the efficacy and safety of newer beta lactams with each other in/outside of urinary tract infections. Therefore, IDSA does not recommend one new beta lactam over the other; however, they recommend cefiderocol as an alternative treatment in infections outside of the urinary tract due to lack of improvement in the outcome as observed in other new beta lactams (despite performing as well as past backbone treatments for DTR-*P. aeruginosa*) [89]. Another important issue is whether it is rational to use new antibiotics in empirical therapy (being selected for use in cases with local epidemiology positive for resistance to traditional antipseudomonal agents/risk factor for MDR/XDR infections) or as a definitive treatment (being chosen after AST results confirming resistance to other antibiotics and susceptibility to the selected new beta lactam).

New Antibiotics

Ceftolozane-tazobactam, a beta-lactam/beta-lactamase inhibitor, is a relatively novel antibiotic showing efficacy in treating *Pseudomonas* infections (urinary tract, intra-abdominal, and pulmonary infections at double doses) (Aspect trials) [115,116,117]. It is less affected by efflux systems and decreased membrane permeability [118]. It has a low affinity for hydrolysis by AmpC, but it is affected by carbapenemases [119], and cases with in vivo resistance have been reported with the main mechanism of action being hyper-expression or the modification of intrinsic AmpC and horizontally acquired beta-lactamases [120]. Even though cases with resistance to ceftolozane-tazobactam have been reported, *P. aeruginosa* isolates are usually susceptible to this drug [121]. 

Ceftazidime-avibactam is a beta-lactam/non-beta-lactam beta-lactamase inhibitor that is not active against metallo-beta-lactamases, and it is affected more by efflux systems and porine changes compared to ceftolozane-tazobactam. Avibactam inhibits the beta-lactamases of class A, KPC, AmpC, and OXA-48. Resistance to KPC was evidenced [108,119], and avibactam is in vitro active also against GES enzymes [108]. The ERACE-PA global study group showed susceptibility to ceftazidime-avibactam of 91% and 72% for carbapenem-susceptible and carbapenem-resistant strains, respectively [121].

Meropenem-vaborbactam is a carbapenem/non-beta-lactam beta-lactamase inhibitor combination showing activities similar to simple meropenem in *P. aeruginosa* infections, as meropenem resistance in *P. aeruginosa* is mostly a result of mechanisms not impacted by vaborbactam. Indeed, vaborbactam inhibits the beta-lactamases of class A and C, while the resistance of *P. aeruginosa* to meropenem is mostly due to efflux systems, reduced membrane permeability, and the beta-lactamases of class B or D [119].

Imipenem-cilastatin-relebactam is another new carbapenem/non-beta-lactam beta-lactamase inhibitor combination. It inhibits the beta lactamases of class A and C but is not active against metallo-beta-lactamases [119]. It was shown to be active for isolates resistant to ceftolozane-tazobactam and ceftazidime-avibactam, making it a valuable option as a rescue therapy [108].

Cefiderocol is a novel siderophore cephalosporin that binds to penicillin-binding proteins, thus preventing the synthesis of the bacterial cell wall. It exploits bacterial iron transporters in order to enter the outer cell membrane. It is poorly affected by efflux systems and porin channel modifications and remains stable against AmpC and metallo-beta-lactamases [119]. Cefiderocol was found to be active against isolates that are resistant to all other newer beta lactams, and it exhibited similar microbiological and clinical efficacy compared to the best available therapy in treating infections due to carbapenem-resistant Gram-negative bacteria (CREDIBLE-CR) [113,122,123].

**Figure 3:**
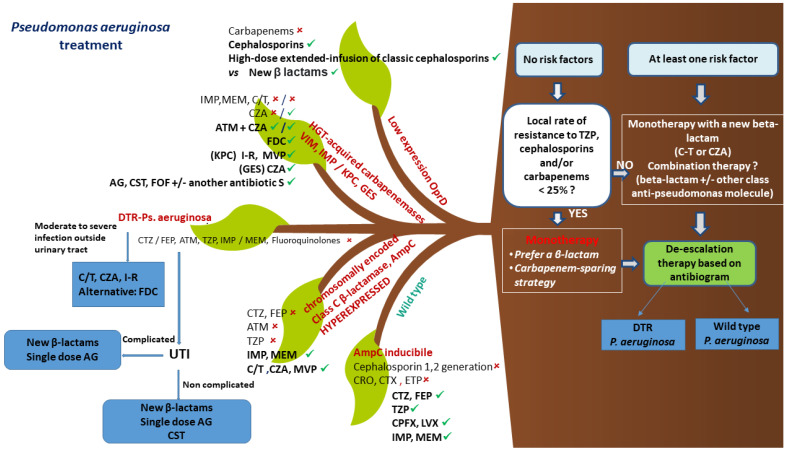
Algorithm for the empirical and targeted treatment of *P. aeruginosa* infections. Abbreviations: AmpC, Ambler class C beta-lactamase; VIM, Verona Integron-encoded Metallo-β-lactamase; IMP, active on imipenem; KPC, Klebsiella pneumonia carbapenemase; GES, Guiana Extended-Spectrum β-lactamase; UTI, urinary tract infections; DTR, difficult to treat resistance; CRO, ceftriaxone; CTX, cefotaxime; ETP, ertapenem; CTZ, ceftazidime; FEP, cefepime; TZP, piperacillin-tazobactam; CPFX, ciprofloxacin; LVX, levofloxacin; IMP, imipenem-cilastatin; MEM, meropenem; ATM, aztreonam; C/T, ceftolozane-tazobactam; CZA, ceftazidime-avibactam; MVP, meropenem-vaborbactam, I-R, imipenem-cilastatin-relebactam; FDC, Cefiderocol; AG, aminoglycosides; CST, colistin; FOF, fosfomycin; OprD, outer membrane porin D; ✓, susceptible; ✕, resistant.

A synoptic flow chart of empirical and targeted treatment for *P. aeruginosa* infections is presented in Figure 3.

## 4. Conclusions

MDR/XDR *P. aeruginosa* is emerging as a major threat related to adverse healthcare consequences. The importance of infection prevention takes on a particular value because it can rapidly develop resistance even to the newest drugs. Moreover, treatment choices should be cautious, sparing newer antibiotics when dealing with a suspected/confirmed sensitive *P. aeruginosa* and choosing the right option for MDR/XDR cases based on specific types and resistance mechanisms. The use of new antibiotics should be rational, both empirically (being selected for use in cases with local epidemiology positive for resistance to traditional antipseudomonal agents/risk factor for MDR/XDR infections) or as definitive treatments (being chosen after AST results confirming resistance to other antibiotics and susceptibility to the selected new beta lactam). Regarding resistance mechanisms, ceftolozane-tazobactam currently shows less vulnerability to common resistance mechanisms, such as efflux systems and reduced membrane permeability, compared to ceftazidime-avibactam. Imipenem-cilastatin-relebactam and cefiderocol are also unaffected by such mechanisms, and studies evidenced that isolates resistant to ceftazidime-avibactam and ceftolozane-tazobactam may remain susceptible to imipenem-cilastatin-relebactam or cefiderocol. Therefore, it is advisable to preserve the use of these two antibiotics in order to exploit them in cases of absolute need.

## Figures and Tables

**Table 1:** *Pseudomonas aeruginosa* resistance based of antibiotic classes.

	Individual Antimicrobials Tested against *P. aeruginosa*		
**Phenotype**	Resistant to at least 1 of the below compounds		
**Carbapenem-resistant**	Imipenem-cilastatin	Meropenem	Doripenem
**Cephalosporin-resistant**	Ceftazidime	Cefepime	
**Fluoroquinolone-resistant**	Ciprofloxacin	Levofloxacin	
**Aminoglycoside-resistant**	Amikacin	Gentamycin	Tobramycin
**Ureido penicillin-resistant**	Piperacillin	Piperacillin-Tazobactam	

**Table 2:** Antipseudomonal agents and the dynamics of their MIC breakpoint change.

Traditional Antibiotics	EUCAST 2019 *		EUCAST 2023 **		New Antibiotics	EUCAST 2019 *		EUCAST 2023 **	
	MIC Breakpoints					MIC Breakpoints			
	S≤	R>	S≤	R>		S≤	R>	S≤	R>
**Beta Lactams**									
Cephalosporin									
Ceftazidime	8	8	0.001	8	Ceftazidime-Avibactam	8	8	8	8
Cefepime	8	8	0.001	8	Ceftolozane-Tazobactam	4	4	4	4
					Cefiderocol			2	2
Ureidopenicillin									
Piperacillin/tazobactam	16	16	0.001	16					
	
Carbapenem									
Imipenem-cilastatin	4	4	0.001	4	Imipenem-Cilastatin-Relebactam				
Meropenem	2	8	2	2/8	Meropenem-Vaborbactam	8	8	8	8
Doripenem			0.001	2					
	
Monobactam									
Aztreonam	16	16	0.001	16	Aztreonam-Avibactam				
Other antibiotics									
Polymixin									
Colistin	2	2	4	4					
	
Fluoroquinolones									
Ciprofloxacin	0.5	0.5	0.001	0.5					
Levofloxacin	1	1	0.001	2					
Aminoglycosides									
Gentamycin	4	4	IE	IE	Plazomicin				
Amikacin	8	16	16	16					
Tobramycin	4	4	2	2					
Fosfomycin									

Abbreviations: EUCAST, European Committee on Antimicrobial Susceptibility Testing; IE, insufficient evidence; S, susceptible; R, resistant. * EUCAST breakpoint when the new intermediate definition was introduced. ** Current EUCAST breakpoints.

**Table 3:** Major resistance mechanism of *P. aeruginosa* based on antibiotics classes.

	Resistance Mechanisms			
**Antibiotic class**	Mechanism 1	Mechanism 2	Mechanism 3	Mechanism 4
**Beta-lactams**	chromosomal AmpC hyper-expression	OprM porin mutation or loss	OXA-1 & -2 enzyme production	MexXY efflux pump overexpression
**Aminoglycosides**	altered permeability	cytoplasm expression of aminoglycoside-modifying enzymes, such as aminoglycoside-2″-O-nucleotidyltransferase ANT (ANT 2″Ia) and aminoglycoside 4′-O-adenylyltransferase (ANT 4′-IIb	overexpression of MexXY efflux pumps	
**Fluoroquinolones**	gyrase (gyr A)—topoisomerase expression; (par C) mutations	altered permeability	efflux systems	
**Carbapenems**	OprD porin loss	MexXY efflux pump expression	beta-lactamase production	

## Data Availability

The information collected for this study is available upon request to the corresponding Author.

## References

[1] World Health Organization (2017). Global Priority List of Antibiotic-Resistant Bacteria to Guide Research, Discovery, and Development of New Antibiotics. http://www.who.int/medicines/publications/WHO-PPL-Short_Summary_25Feb-ET_NM_WHO.pdf.

[2] Rice L.B. (2008). Federal Funding for the Study of Antimicrobial Resistance in Nosocomial Pathogens: No ESKAPE. J. Infect. Dis..

[3] Suetens C., Latour K., Kärki T., Ricchizzi E., Kinross P., Moro M.L., Jans B., Hopkins S., Hansen S., Lyytikäinen O. (2018). Prevalence of healthcare-associated infections, estimated incidence and composite antimicrobial resistance index in acute care hospitals and long-term care facilities: Results from two European point prevalence surveys, 2016 to 2017. Eurosurveillance.

[4] Vincent J.-L., Sakr Y., Singer M., Martin-Loeches I., Machado F.R., Marshall J.C., Finfer S., Pelosi P., Brazzi L., Aditianingsih D. (2020). Prevalence and Outcomes of Infection Among Patients in Intensive Care Units in 2017. JAMA.

[5] Ribeiro C.D.S., Crozatti M.T.L., da Silva A.A., Macedo R.S., Machado A.M.D.O., Silva A.T.D.A. (2020). Pseudomonas aeruginosa in the ICU: Prevalence, resistance profile, and antimicrobial consumption. Rev. Soc. Bras. Med. Trop..

[6] Centers for Disease Control and Prevention (2019). Antibiotic Resistance Threats in the United States. https://www.cdc.gov/drugresistance/pdf/threats-report/2019-ar-threats-report-508.pd.

[7] Nathwani D., Raman G., Sulham K., Gavaghan M., Menon V. (2014). Clinical and economic consequences of hospital-acquired resistant and multidrug-resistant Pseudomonas aeruginosa infections: A systematic review and meta-analysis. Antimicrob. Resist. Infect. Control.

[8] Hirsch E.B., Tam V.H. (2010). Impact of multidrug-resistant *Pseudomonas aeruginosa* infection on patient outcomes. Expert Rev. Pharm. Outcomes Res..

[9] Matos E.C.O.D., Andriolo R.B., Rodrigues Y.C., Lima P.D.L.D., Carneiro I.C.D.R.S., Lima K.V.B. (2018). Mortality in patients with multidrug-resistant Pseudomonas aeruginosa infections: A meta-analysis. Rev. Soc. Bras. Med. Trop..

[10] Magiorakos A.-P., Srinivasan A., Carey R.B., Carmeli Y., Falagas M.E., Giske C.G., Harbarth S., Hindler J.F., Kahlmeter G., Olsson-Liljequist B. (2012). Multidrug-resistant, extensively drug-resistant and pandrug-resistant bacteria: An international expert proposal for interim standard definitions for acquired resistance. Clin. Microbiol. Infect..

[11] Kadri S.S., Adjemian J., Lai Y.L., Spaulding A.B., Ricotta E., Prevots D.R., Palmore T.N., Rhee C., Klompas M., Dekker J.P. (2018). Difficult-to-Treat Resistance in Gram-negative Bacteremia at 173 US Hospitals: Retrospective Cohort Analysis of Prevalence, Predictors, and Outcome of Resistance to All First-line Agents. Clin. Infect. Dis..

[12] ECDC (2022). Antimicrobial Resistance Surveillance in EUROPE 2020 Data. https://www.ecdc.europa.eu/sites/default/files/documents/Joint-WHO-ECDC-AMR-report-2022.pdf.

[13] Reynolds D., Kollef M. (2021). The Epidemiology and Pathogenesis and Treatment of Pseudomonas aeruginosa Infections: An Update. Drugs.

[14] Morin C.D., Déziel E., Gauthier J., Levesque R.C., Lau G.W. (2021). An Organ System-Based Synopsis of *Pseudomonas aeruginosa* Virulence. Virulence.

[15] Durante-Mangoni E., Andini R., Agrusta F., Iossa D., Mattucci I., Bernardo M., Utili R. (2014). Infective endocarditis due to multidrug resistant gram-negative bacilli: Single centre experience over 5years. Eur. J. Intern. Med..

[16] Falcone M., Tiseo G., Durante-Mangoni E., Ravasio V., Barbaro F., Ursi M.P., Pasticci M.B., Bassetti M., Grossi P., Venditti M. (2018). Risk Factors and Outcomes of Endocarditis Due to Non-HACEK Gram-Negative Bacilli: Data from the Prospective Multicenter Italian Endocarditis Study Cohort. Antimicrob. Agents Chemother..

[17] Hombach M., Zbinden R., Böttger E.C. (2013). Standardisation of disk diffusion results for antibiotic susceptibility testing using the sirscan automated zone reader. BMC Microbiol..

[18] Clinical and Laboratory Standards Institute Performance and Standards for Antimicrobial Susceptibility Testing. https://www.nih.org.pk/wp-content/uploads/2021/02/CLSI-2020.pdf.

[19] Lavano M.A., Foglia F., Della Rocca M.T., Folliero V., Zannella C., Crudele V., Boccia G., Franci G., Finamore E., Galdiero M. (2020). Epidemiology of Multi and Extensively Drug Resistance Pseudomonas Aeruginosa Infections from University Hospital “Luigi Vanvitelli”. J. Community Med. Public Health.

[20] European Committee on Antimicrobial Susceptibility Testing. https://www.eucast.org/fileadmin/src/media/PDFs/EUCAST_files/Breakpoint_tables/v_13.0_Breakpoint_Tables.pdf.

[21] Daragon B., Fournier D., Plésiat P., Jeannot K. (2021). Performance of disc diffusion, MIC gradient tests and Vitek 2 for ceftolozane/tazobactam and ceftazidime/avibactam susceptibility testing of *Pseudomonas aeruginosa*. J. Antimicrob. Chemother..

[22] Dwivedi H.P., Franklin S., Chandrasekaran S., Garner O., Traczewski M.M., Beasley D., Procop G.W., Tuohy M., Wilson D., Bala Y. (2022). Multicenter Clinical Evaluation of Vitek 2 Meropenem-Vaborbactam for Susceptibility Testing of Enterobacterales and *Pseudomonas aeruginosa*. J. Clin. Microbiol..

[23] Jean S., Garrett S., Anglade C., Bridon L., Davies L., Garner O.B., Richards J., Wallace M., Wootton M., Burnham C.-A.D. (2019). Multicenter Clinical Evaluation of Etest Meropenem-Vaborbactam (bioMérieux) for Susceptibility Testing of *Enterobacterales* (*Enterobacteriaceae*) and Pseudomonas aeruginosa. J. Clin. Microbiol..

[24] Devoos L., Biguenet A., Rousselot J., Bour M., Plésiat P., Fournier D., Jeannot K. (2022). Performance of discs, sensititre EUMDROXF microplates and MTS gradient strips for the determination of the susceptibility of multidrug-resistant Pseudomonas aeruginosa to cefiderocol. Clin. Microbiol. Infect..

[25] Tan T.Y., Ng S.Y. (2007). Comparison of Etest, Vitek and agar dilution for susceptibility testing of colistin. Clin. Microbiol. Infect..

[26] Matuschek E., Åhman J., Webster C., Kahlmeter G. (2018). Antimicrobial susceptibility testing of colistin—Evaluation of seven commercial MIC products against standard broth microdilution for Escherichia coli, Klebsiella pneumoniae, Pseudomonas aeruginosa, and Acinetobacter spp.. Clin. Microbiol. Infect..

[27] European Committee on Antimicrobial Susceptibility Testing On Recent Changes in Clinical Microbiology Susceptibility Reports—New Interpretation of Susceptibility Categories S, I and R. https://www.eucast.org/fileadmin/src/media/PDFs/EUCAST_files/Guidance_documents/To_clinical_colleagues_on_recent_changes_in_clinical_microbiology_susceptibility_reports_9_July2021.pdf.

[28] The European Committee on Antimicrobial Susceptibility Testing (2019). Clinical Breakpoints—Bacteria (v 9.0). https://www.eucast.org/fileadmin/src/media/PDFs/EUCAST_files/Breakpoint_tables/v_9.0_Breakpoint_Tables.pdf.

[29] Davey M.E., O’Toole G.A. (2000). Microbial Biofilms: From Ecology to Molecular Genetics. Microbiol. Mol. Biol. Rev..

[30] Rasamiravaka T., Labtani Q., Duez P., El Jaziri M. (2015). The Formation of Biofilms by Pseudomonas aeruginosa: A Review of the Natural and Synthetic Compounds Interfering with Control Mechanisms. Biomed Res. Int..

[31] Ghafoor A., Hay I.D., Rehm B.H.A. (2011). Role of Exopolysaccharides in Pseudomonas aeruginosa Biofilm Formation and Architecture. Appl. Environ. Microbiol..

[32] Leid J.G. (2009). Bacterial biofilms resist key host defenses. Microbe.

[33] Page M.G., Heim J. (2009). Prospects for the next anti-Pseudomonas drug. Curr. Opin. Pharmacol..

[34] Watnick P., Kolter R. (2000). Biofilm, city of microbes. J. Bacteriol..

[35] Flemming H.-C., Neu T.R., Wozniak D.J. (2007). The EPS matrix: The “house of biofilm cells”. J. Bacteriol..

[36] Donlan R.M., Costerton J.W. (2002). Biofilms: Survival Mechanisms of Clinically Relevant Microorganisms. Clin. Microbiol. Rev..

[37] Ghannoum M., O’Toole G.A. (2004). Microbial Biofilms.

[38] Haussler S., Fuqua C. (2013). Biofilms 2012: New Discoveries and Significant Wrinkles in a Dynamic Field. J. Bacteriol..

[39] Castro J., França A., Bradwell K.R., Serrano M.G., Jefferson K.K., Cerca N. (2017). Comparative transcriptomic analysis of Gardnerella vaginalis biofilms vs. planktonic cultures using RNA-seq. NPJ Biofilms Microbiomes.

[40] Nielsen S.M., Penstoft L.N., Nørskov-Lauritsen N. (2019). Motility, Biofilm Formation and Antimicrobial Efflux of Sessile and Planktonic Cells of Achromobacter xylosoxidans. Pathogens.

[41] Hoyle B.D., Costerton J.W. (1991). Bacterial resistance to antibiotics: The role of biofilms. Prog. Drug Res..

[42] Folliero V., Franci G., Dell’Annunziata F., Giugliano R., Foglia F., Sperlongano R., De Filippis A., Finamore E., Galdiero M. (2021). Evaluation of Antibiotic Resistance and Biofilm Production among Clinical Strain Isolated from Medical Devices. Int. J. Microbiol..

[43] Neopane P., Nepal H.P., Shrestha R., Uehara O., Abiko Y. (2018). In vitro biofilm formation by Staphylococcus aureus isolated from wounds of hospital-admitted patients and their association with antimicrobial resistance. Int. J. Gen. Med..

[44] Vuotto C., Longo F., Pascolini C., Donelli G., Balice M., Libori M., Tiracchia V., Salvia A., Varaldo P. (2017). Biofilm formation and antibiotic resistance in *Klebsiella pneumoniaeurinary* strains. J. Appl. Microbiol..

[45] Hassan A., Usman J., Kaleem F., Omair M., Khalid A., Iqbal M. (2011). Evaluation of different detection methods of biofilm formation in the clinical isolates. Braz. J. Infect. Dis..

[46] Lima J.L.D.C., Alves L.R., Da Paz J.N.P., Rabelo M.A., Maciel M.A.V., De Morais M.M.C. (2017). Analysis of biofilm production by clinical isolates of Pseudomonas aeruginosa from patients with ventilator-associated pneumonia. Rev. Bras. Ter. Intensiv..

[47] Silva N., Marques L., Röder D. (2021). Diagnosis of biofilm infections: Current methods used, challenges and perspectives for the future. J. Appl. Microbiol..

[48] Thi M., Wibowo D., Rehm B. (2020). Pseudomonas aeruginosa Biofilms. Int. J. Mol. Sci..

[49] Gellatly S.L., Hancock R.E. (2013). Pseudomonas aeruginosa: New insights into pathogenesis and host defenses. Pathog. Dis..

[50] Restrepo M.I., Babu B.L., Reyes L.F., Chalmers J.D., Soni N.J., Sibila O., Faverio P., Cilloniz C., Rodriguez-Cintron W., Aliberti S. (2018). Burden and risk factors for *Pseudomonas aeruginosa* community-acquired pneumonia: A multinational point prevalence study of hospitalised patients. Eur. Respir. J..

[51] Rello J., Allegri C., Rodriguez A., Vidaur L., Sirgo G., Gomez F., Agbaht K., Pobo A., Diaz E. (2006). Risk Factors for Ventilator-associated Pneumonia by *Pseudomonas aeruginosa* in Presence of Recent Antibiotic Exposure. Anesthesiology.

[52] Parker C.M., Kutsogiannis J., Muscedere J., Cook D., Dodek P., Day A.G., Heyland D.K. (2008). Ventilator-associated pneumonia caused by multidrug-resistant organisms or Pseudomonas aeruginosa: Prevalence, incidence, risk factors, and outcomes. J. Crit. Care.

[53] Raman G., Avendano E.E., Chan J., Merchant S., Puzniak L. (2018). Risk factors for hospitalized patients with resistant or multidrug-resistant Pseudomonas aeruginosa infections: A systematic review and meta-analysis. Antimicrob. Resist. Infect. Control.

[54] Merchant S., Proudfoot E.M., Quadri H.N., McElroy H.J., Wright W.R., Gupta A., Sarpong E.M. (2018). Risk factors for Pseudomonas aeruginosa infections in Asia-Pacific and consequences of inappropriate initial antimicrobial therapy: A systematic literature review and meta-analysis. J. Glob. Antimicrob. Resist..

[55] Aloush V., Navon-Venezia S., Seigman-Igra Y., Cabili S., Carmeli Y. (2006). Multidrug-Resistant *Pseudomonas aeruginosa*: Risk Factors and Clinical Impact. Antimicrob. Agents Chemother..

[56] Paramythiotou E., Lucet J.-C., Timsit J.-F., Vanjak D., Paugam-Burtz C., Trouillet J.-L., Belloc S., Kassis N., Karabinis A., Andremont A. (2004). Acquisition of multidrug-resistant Pseudomonas aeruginosa in patients in intensive care units: Role of antibiotics with antipseudomonal activity. Clin. Infect. Dis..

[57] Lieberman D., Lieberman D. (2003). Pseudomonal infections in patients with COPD: Epidemiology and management. Am. J. Respir. Med..

[58] Harris A.D., Smith D., Johnson J.A., Bradham D.D., Roghmann M.-C. (2002). Risk Factors for Imipenem-Resistant Pseudomonas aeruginosa among Hospitalized Patients. Clin. Infect. Dis..

[59] Montero M.M., Sala M., Riú M., Belvis F., Salvado M., Grau S., Horcajada J.P., Alvarez-Lerma F., Terradas R., Orozco-Levi M. (2009). Risk factors for multidrug-resistant Pseudomonas aeruginosa acquisition. Impact of antibiotic use in a double case–control study. Eur. J. Clin. Microbiol. Infect. Dis..

[60] Palacios-Baena Z.R., Giannella M., Manissero D., Rodríguez-Baño J., Viale P., Lopes S., Wilson K., McCool R., Longshaw C. (2020). Risk factors for carbapenem-resistant Gram-negative bacterial infections: A systematic review. Clin. Microbiol. Infect..

[61] Patolia S., Abate G., Patel N., Patolia S., Frey S. (2017). Risk factors and outcomes for multidrug-resistant Gram-negative bacilli bacteremia. Ther. Adv. Infect. Dis..

[62] Al Hamdan A.S., Alghamdi A.A., Alyousif G.F., Hamza F.A., Shafey M.M., AlAmri A.M., Sunki A.A. (2022). Evaluating the Prevalence and the Risk Factors of Gram-Negative Multi-Drug Resistant Bacteria in Eastern Saudi Arabia. Infect. Drug Resist..

[63] Karruli A., Boccia F., Gagliardi M., Patauner F., Ursi M.P., Sommese P., De Rosa R., Murino P., Ruocco G., Corcione A. (2021). Multidrug-Resistant Infections and Outcome of Critically Ill Patients with Coronavirus Disease 2019: A Single Center Experience. Microb. Drug Resist..

[64] Karruli A., de Cristofaro J., Andini R., Iossa D., Bernardo M., Amarelli C., Mattucci I., Zampino R., Zarrilli R., Durante-Mangoni E. (2021). Risk Factors and Outcome of Multidrug-Resistant Infections after Heart Transplant: A Contemporary Single Center Experience. Microorganisms.

[65] Spagnolo A.M., Sartini M., Cristina M.L. (2021). Pseudomonas aeruginosa in the healthcare facility setting. Rev. Med. Microbiol..

[66] Morita Y., Tomida J., Kawamura Y. (2014). Responses of Pseudomonas aeruginosa to antimicrobials. Front. Microbiol..

[67] Livermore D.M. (2002). Multiple Mechanisms of Antimicrobial Resistance in Pseudomonas aeruginosa: Our Worst Nightmare?. Clin. Infect. Dis..

[68] Sharma G., Rao S., Bansal A., Dang S., Gupta S., Gabrani R. (2014). Pseudomonas aeruginosa biofilm: Potential therapeutic targets. Biologicals.

[69] Pachori P., Gothalwal R., Gandhi P. (2019). Emergence of antibiotic resistance Pseudomonas aeruginosa in intensive care unit; a critical review. Genes Dis..

[70] Henrichfreise B., Wiegand I., Pfister W., Wiedemann B. (2007). Resistance Mechanisms of Multiresistant *Pseudomonas aeruginosa* Strains from Germany and Correlation with Hypermutation. Antimicrob. Agents Chemother..

[71] Pang Z., Raudonis R., Glick B.R., Lin T.J., Cheng Z. (2019). Antibiotic resistance in Pseudomonas aeruginosa: Mechanisms and alternative therapeutic strategies. Biotechnol. Adv..

[72] Bert F., Branger C., Lambert-Zechovsky N. (2002). Identification of PSE and OXA beta-lactamase genes in Pseudomonas aeruginosa using PCR-restriction fragment length polymorphism. J. Antimicrob. Chemother..

[73] Ramirez M.S., Tolmasky M.E. (2010). Aminoglycoside modifying enzymes. Drug Resist. Updat..

[74] Memar M.Y., Pormehrali R., Alizadeh N., Ghotaslou R., Baghi H.B. (2016). Colistin, an option for treatment of multiple drug resistant Pseudomonas aeruginosa. Physiol. Pharmacol..

[75] Iregui M., Ward S., Sherman G., Fraser V.J., Kollef M.H. (2002). Clinical Importance of Delays in the Initiation of Appropriate Antibiotic Treatment for Ventilator-Associated Pneumonia. Chest.

[76] Mesaros N., Nordmann P., Plésiat P., Roussel-Delvallez M., Van Eldere J., Glupczynski Y., Van Laethem Y., Jacobs F., Lebecque P., Malfroot A. (2007). Pseudomonas aeruginosa: Resistance and therapeutic options at the turn of the new millennium. Clin. Microbiol. Infect..

[77] Bassetti M., Vena A., Croxatto A., Righi E., Guery B. (2018). How to manage Pseudomonas aeruginosa infections. Drugs Context.

[78] Paul M., Leibovici L. (2013). Editorial commentary: Combination therapy for Pseudomonas aeruginosa bacteremia: Where do we stand?. Clin. Infect. Dis..

[79] Thu P.N.T., Huong M.N.T., Thi N.T., Thanh H.N., Minh K.P. (2021). Combination antibiotic therapy versus monotherapy in the treatment of acute exacerbations of chronic obstructive pulmonary disease: An open-label randomized trial. BMC Infect. Dis..

[80] Babich T., Naucler P., Valik J.K., Giske C.G., Benito N., Cardona R., Rivera A., Pulcini C., Fattah M.A., Haquin J. (2021). Combination versus monotherapy as definitive treatment for *Pseudomonas aeruginosa* bacteraemia: A multicentre retrospective observational cohort study. J. Antimicrob. Chemother..

[81] Park S.-Y., Park H.J., Moon S.M., Park K.-H., Chong Y.P., Kim M.-N., Kim S.-H., Lee S.-O., Kim Y.S., Woo J.H. (2012). Impact of adequate empirical combination therapy on mortality from bacteremic Pseudomonas aeruginosa pneumonia. BMC Infect. Dis..

[82] Ibrahim D., Jabbour J.-F., Kanj S.S. (2020). Current choices of antibiotic treatment for Pseudomonas aeruginosa infections. Curr. Opin. Infect. Dis..

[83] Tamma P.D., Aitken S.L., Bonomo R.A., Mathers A.J., van Duin D., Clancy C.J. (2020). Infectious Diseases Society of America Guidance on the Treatment of Extended-Spectrum β-lactamase Producing Enterobacterales (ESBL-E), Carbapenem-Resistant Enterobacterales (CRE), and *Pseudomonas aeruginosa* with Difficult-to-Treat Resistance (DTR-*P. aeruginosa*). Clin. Infect. Dis..

[84] Farhan S.M., Raafat M., Abourehab M.A.S., El-Baky R.M.A., Abdalla S., El-Gendy A.O., Azmy A.F. (2021). Effect of Imipenem and Amikacin Combination against Multi-Drug Resistant *Pseudomonas aeruginosa*. Antibiotics.

[85] Yadav R., Bulitta J.B., Nation R.L., Landersdorfer C.B. (2017). Optimization of Synergistic Combination Regimens against Carbapenem- and Aminoglycoside-Resistant Clinical Pseudomonas aeruginosa Isolates via Mechanism-Based Pharmacokinetic/Pharmacodynamic Modeling. Antimicrob. Agents Chemother..

[86] Deelen J.T., Rottier W., Buiting A., Dorigo-Zetsma J., Kluytmans J., van der Linden P., Thijsen S., Vlaminckx B., Weersink A., Ammerlaan H. (2021). Short-course aminoglycosides as adjunctive empirical therapy in patients with Gram-negative bloodstream infection, a cohort study. Clin. Microbiol. Infect..

[87] Papst L., Beović B., Pulcini C., Durante-Mangoni E., Rodríguez-Baño J., Kaye K.S., Daikos G.L., Raka L., Paul M., on behalf ofESGAP, ESGBIS, ESGIE and the CRGNB Treatment Survey Study Group (2018). Antibiotic treatment of infections caused by carbapenem-resistant Gram-negative bacilli: An international ESCMID cross-sectional survey among infectious diseases specialists practicing in large hospitals. Clin. Microbiol. Infect..

[88] Coppola N., Maraolo A.E., Onorato L., Scotto R., Calò F., Atripaldi L., Borrelli A., Corcione A., De Cristofaro M.G., Durante-Mangoni E. (2022). Epidemiology, Mechanisms of Resistance and Treatment Algorithm for Infections Due to Carbapenem-Resistant Gram-Negative Bacteria: An Expert Panel Opinion. Antibiotics.

[89] Tamma P.D., Aitken S.L., Bonomo R.A., Mathers A.J., van Duin D., Clancy C.J. (2022). Infectious Diseases Society of America 2022 Guidance on the Treatment of Extended-Spectrum β-lactamase Producing Enterobacterales (ESBL-E), Carbapenem-Resistant Enterobacterales (CRE), and *Pseudomonas aeruginosa* with Difficult-to-Treat Resistance (DTR-*P. aeruginosa*). Clin. Infect. Dis..

[90] Young O., Psirides A. (2020). Wellington ICU Drug Manual. https://drug.wellingtonicu.com/PDF/WellingtonICUDrugManual.pdf.

[91] Köhler T., Michea-Hamzehpour M., Plesiat P., Kahr A.L., Pechere J.C. (1997). Differential selection of multi drug efflux systems by quinolones in Pseudomonas aeruginosa. Antimicrob. Agents Chemother..

[92] Durante-Mangoni E., Grammatikos A., Utili R., Falagas M.E. (2009). Do we still need the aminoglycosides?. Int. J. Antimicrob. Agents.

[93] Lahiri T., Hempstead S.E., Brady C., Cannon C.L., Clark K., Condren M.E., Guill M.F., Guillerman R.P., Leone C.G., Maguiness K. (2016). Clinical Practice Guidelines From the Cystic Fibrosis Foundation for Preschoolers with Cystic Fibrosis. Pediatrics.

[94] Shaeer K.M., Zmarlicka M.T., Chahine E.B., Piccicacco N., Cho J.C. (2019). Plazomicin: A next-generation aminoglycoside. Pharmacother. J. Hum. Pharmacol. Drug Ther..

[95] Durante-Mangoni E., Andini R., Signoriello S., Cavezza G., Murino P., Buono S., De Cristofaro M., Taglialatela C., Bassetti M., Malacarne P. (2016). Acute kidney injury during colistin therapy: A prospective study in patients with extensively-drug resistant Acinetobacter baumannii infections. Clin. Microbiol. Infect..

[96] Florescu D.F., Qiu F., McCartan M.A., Mindru C., Fey P.D., Kalil A.C. (2012). What Is the Efficacy and Safety of Colistin for the Treatment of Ventilator-Associated Pneumonia? A Systematic Review and Meta-Regression. Clin. Infect. Dis..

[97] Boisson M., Jacobs M., Grégoire N., Gobin P., Marchand S., Couet W., Mimoz O. (2014). Comparison of intrapulmonary and systemic pharmacokinetics of colistin methanesulfonate (CMS) and colistin after aerosol delivery and intravenous administration of CMS in critically ill patients. Antimicrob. Agents Chemother..

[98] Zak-Doron Y., Benattar Y.D., Pfeffer I., Daikos G.L., Skiada A., Antoniadou A., Durante-Mangoni E., Andini R., Cavezza G., Leibovici L. (2018). The Association Between Empirical Antibiotic Treatment and Mortality in Severe Infections Caused by Carbapenem-resistant Gram-negative Bacteria: A Prospective Study. Clin. Infect. Dis..

[99] Nutman A., Lellouche J., Temkin E., Daikos G., Skiada A., Durante-Mangoni E., Dishon-Benattar Y., Bitterman R., Yahav D., Daitch V. (2020). Colistin plus meropenem for carbapenem-resistant Gram-negative infections: In vitro synergism is not associated with better clinical outcomes. Clin. Microbiol. Infect..

[100] Paul M., Daikos G.L., Durante-Mangoni E., Yahav D., Carmeli Y., Benattar Y.D., Skiada A., Andini R., Eliakim-Raz N., Nutman A. (2018). Colistin alone versus colistin plus meropenem for treatment of severe infections caused by carbapenem-resistant Gram-negative bacteria: An open-label, randomised controlled trial. Lancet Infect. Dis..

[101] Dickstein Y., Leibovici L., Yahav D., Eliakim-Raz N., Daikos G.L., Skiada A., Antoniadou A., Carmeli Y., Nutman A., Levi I. (2016). Multicentre open-label randomised controlled trial to compare colistin alone with colistin plus meropenem for the treatment of severe infections caused by carbapenem-resistant Gram-negative infections (AIDA): A study protocol. BMJ Open.

[102] Giannouli M., Di Popolo A., Durante-Mangoni E., Bernardo M., Cuccurullo S., Amato G., Tripodi M.-F., Triassi M., Utili R., Zarrilli R. (2012). Molecular epidemiology and mechanisms of rifampicin resistance in Acinetobacter baumannii isolates from Italy. Int. J. Antimicrob. Agents.

[103] MONUROL (2007). Prescribing Information.

[104] Reffert J.L., Smith W.J. (2014). Fosfomycin for the Treatment of Resistant Gram-Negative Bacterial Infections. Pharmacother. J. Hum. Pharmacol. Drug Ther..

[105] Mirakhur A., Gallagher M., Ledson M., Hart C., Walshaw M. (2003). Fosfomycin therapy for multiresistant *Pseudomonas aeruginosa* in cystic fibrosis. J. Cyst. Fibros..

[106] Asuphon O., Montakantikul P., Houngsaitong J., Kiratisin P., Sonthisombat P. (2016). Optimizing intravenous fosfomycin dosing in combination with carbapenems for treatment of Pseudomonas aeruginosa infections in critically ill patients based on pharmacokinetic/pharmacodynamic (PK/PD) simulation. Int. J. Infect. Dis..

[107] Kaye K.S., Rice L.B., Dane A.L., Stus V., Sagan O., Fedosiuk E., Das A.F., Skarinsky D., Eckburg P.B., Ellis-Grosse E.J. (2019). Fosfomycin for Injection (ZTI-01) Versus Piperacillin-tazobactam for the Treatment of Complicated Urinary Tract Infection Including Acute Pyelonephritis: ZEUS, A Phase 2/3 Randomized Trial. Clin. Infect. Dis..

[108] Coyne A.J.K., El Ghali A., Holger D., Rebold N., Rybak M.J. (2022). Therapeutic Strategies for Emerging Multidrug-Resistant Pseudomonas aeruginosa. Infect. Dis. Ther..

[109] Bauer K.A., West J.E., O’Brien J.M., Goff D.A. (2013). Extended-Infusion Cefepime Reduces Mortality in Patients with Pseudomonas aeruginosa Infections. Antimicrob. Agents Chemother..

[110] Lodise T.P., Lomaestro B., Drusano G.L. (2007). Piperacillin-Tazobactam for Pseudomonas aeruginosa Infection: Clinical Implications of an Extended-Infusion Dosing Strategy. Clin. Infect. Dis..

[111] Hong D.J., Bae I.K., Jang I.-H., Jeong S.H., Kang H.-K., Lee K. (2015). Epidemiology and Characteristics of Metallo-β-Lactamase-Producing *Pseudomonas aeruginosa*. Infect. Chemother..

[112] Glen K.A., Lamont I.L. (2021). β-lactam Resistance in *Pseudomonas aeruginosa*: Current Status, Future Prospects. Pathogens.

[113] Karruli A., Massa A., Andini R., Marrazzo T., Ruocco G., Zampino R., Durante-Mangoni E. (2023). Clinical efficacy and safety of cefiderocol for resistant Gram-negative infections: A real-life, single-centre experience. Int. J. Antimicrob. Agents.

[114] Mauri C., Maraolo A.E., Di Bella S., Luzzaro F., Principe L. (2021). The Revival of Aztreonam in Combination with Avibactam against Metallo-β-Lactamase-Producing Gram-Negatives: A Systematic Review of In Vitro Studies and Clinical Cases. Antibiotics.

[115] Solomkin J., Hershberger E., Miller B., Popejoy M., Friedland I., Steenbergen J., Yoon M., Collins S., Yuan G., Barie P.S. (2015). Ceftolozane/tazobactam plus metronidazole for complicated intra-abdominal infections in an era of multidrug resistance: Results from a randomized, double-blind, phase 3 trial (ASPECT-cIAI). Clin. Infect. Dis..

[116] Wagenlehner F.M., Umeh O., Steenbergen J., Yuan G., Darouiche R.O., Wagenlehner F.M., Umeh O., Steenbergen J., Yuan G., Darouiche R.O. (2015). Ceftolozane-tazobactam compared with levofloxacin in the treatment of complicated urinary-tract infections, including pyelonephritis: A randomised, double-blind, phase 3 trial (ASPECT-cUTI). Lancet.

[117] Kollef M.H., Nováček M., Kivistik U., Réa-Neto A., Shime N., Martin-Loeches I., Timsit J.-F., Wunderink R.G., Bruno C.J., Huntington J.A. (2019). Ceftolozane–tazobactam versus meropenem for treatment of nosocomial pneumonia (ASPECT-NP): A randomised, controlled, double-blind, phase 3, non-inferiority trial. Lancet Infect. Dis..

[118] Gallagher J.C., Satlin M.J., Elabor A., Saraiya N., McCreary E.K., Molnar E., El-Beyrouty C., Jones B.M., Dixit D., Heil E.L. (2018). Ceftolozane-Tazobactam for the Treatment of Multidrug-Resistant Pseudomonas aeruginosa Infections: A Multicenter Study. Open Forum Infect. Dis..

[119] Parisio E.M. Camarlinghi G Le Nuove Molecole Antibiotiche per il Trattamento Delle Infezioni da Batteri Gram-Negativi. https://www.infezioniobiettivozero.info/9-infection-control/125-le-nuove-molecole-antibiotiche-per-il-trattamento-delle-infezioni-da-batteri-gram-negativi.

[120] Fraile-Ribot P.A., Cabot G., Mulet X., Periañez L., Martín-Pena M.L., Juan C., Pérez J.L., Oliver A. (2017). Mechanisms leading to in vivo ceftolozane/tazobactam resistance development during the treatment of infections caused by MDR Pseudomonas aeruginosa. J. Antimicrob. Chemother..

[121] Gill C.M., Aktaþ E., Alfouzan W., Bourassa L., Brink A., Burnham C.-A.D., Canton R., Carmeli Y., Falcone M., Kiffer C. (2021). The ERACE-PA Global Surveillance Program: Ceftolozane/tazobactam and Ceftazidime/avibactam in vitro Activity against a Global Collection of Carbapenem-resistant Pseudomonas aeruginosa. Eur. J. Clin. Microbiol. Infect. Dis..

[122] Bassetti M., Echols R., Matsunaga Y., Ariyasu M., Doi Y., Ferrer R., Lodise T.P., Naas T., Niki Y., Paterson D.L. (2021). Efficacy and safety of cefiderocol or best available therapy for the treatment of serious infections caused by carbapenem-resistant Gram-negative bacteria (CREDIBLE-CR): A randomised, open-label, multicentre, pathogen-focused, descriptive, phase 3 trial. Lancet Infect. Dis..

[123] Karruli A., Massa A., Bertolino L., Andini R., Sansone P., Dongiovanni S., Pace M.C., Pota V., Durante-Mangoni E. (2022). Clinical Characteristics and Outcome of MDR/XDR Bacterial Infections in a Neuromuscular Semi-Intensive/Sub-Intensive Care Unit. Antibiotics.

